# Interview with Luc Tytgat, EASA Director, Strategy and Safety Management Directorate

**DOI:** 10.1007/s13272-022-00618-x

**Published:** 2022-11-04

**Authors:** Jean-Pierre Sanfourche, Markus Fischer

**Affiliations:** 1 Deutsches Zentrum für Luft- und Raumfahrt e.V. (DLR), German Aerospace Center, Cologne, Germany; 2 Council of European Aerospace Societies (CEAS), Brussels, Belgium

## Mr Tytgat, we are very pleased to have this opportunity to exchange with you on the EASA role in Research and Innovation. Would you have a word of introduction for our readers?

It is a great pleasure to present to CEAS Aeronautical Journal’s readers the EASA role in research and innovation. As we all know, research and innovation are key for a competitive EU aviation industry, but they also pose a lot of challenges for EASA as regulator and certifying authority. New concepts and technologies bring new scientific, technical and societal challenges. We need to be an integral part of the technological developments from the very beginning to ensure a high safety level and support the growth of the European industry. Aviation is transforming substantially and faster than ever, and we need our competences to grow as an organisation by participating in research projects and setting up strong partnerships with national regulators, research centres, innovation networks and key players in this dynamic arena.
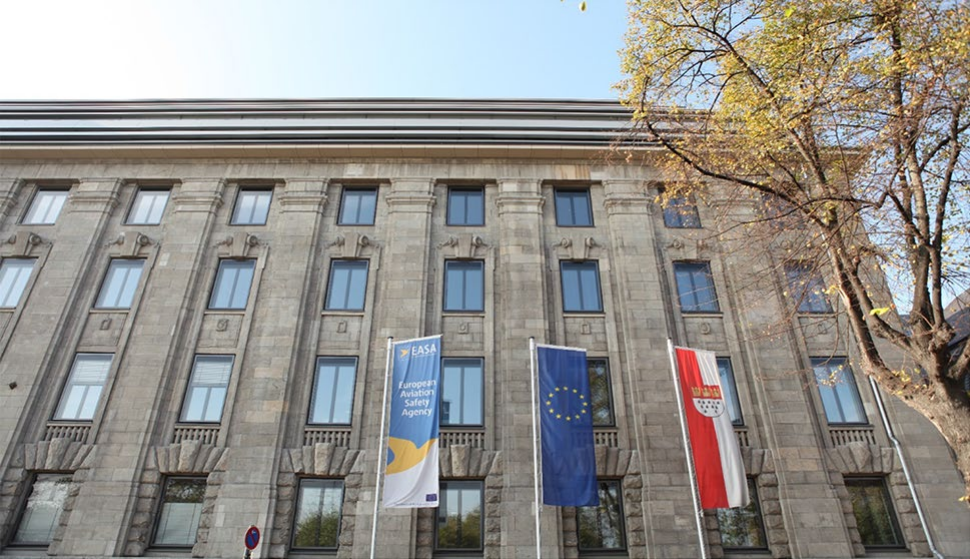


## What is EASA’s role in research and how has it evolved? How are EASA Research & Innovation activities coordinated with ACARE, the European Commission (DG MOVE), SESAR, EUROCONTROL, CLEAN AVIATION, and the EASA Member States’ National Aviation Authorities?

Regulation (EU) 2018/1139, EASA’s Basic Regulation, introduced several new key competences for the Agency. In the field of aviation Research & Innovation (R&I), and more specifically in the strategic areas of safety, security, environmental protection, and more recently, health safety, EASA assists the Commission and the Member States in identifying main research themes, contributing to ensure consistency and coordination between publicly funded research and development. We support the Commission in the definition and accomplishment of the relevant European Union framework programmes for R&I activities and of the annual and multi-annual work programmes, such as Horizon Europe.

Furthermore, as of earlier this year, EASA participates in the new European Public–Private Partnerships Clean Aviation and SESAR 3 Joint Undertakings, providing advice as third party. We dedicate resources to manage numerous delegated budgets and R&I projects under Contribution Agreements with the Commission. We provide technical advice to innovative industry projects carried out through Innovation Partnership Contracts and we assist in submitting solid proposals. With the Member States, we have set up an Advisory Body dedicated to Research and Innovation with a view to developing joint research agendas. We started last year to engage with academia to create the EASA Scientific Committee for sharing and discussing knowledge of advanced scientific developments and have launched a scheme to attract PhD students to share their work with us. The first event related to this activity is planned for March 2023.

EASA is also actively involved in preparing tomorrow’s rules and guidance to accompany the emergence of new technologies in aviation. One noticeable effort is linked to the rapid deployment of Artificial Intelligence (AI) solutions in safety-related applications across all aviation domains. The EASA AI Roadmap represents an action plan involving all impacted aviation stakeholders. Close cooperation with EUROCONTROL on the selection and development of use cases in the ATM domain provides EASA with means to test the guidance that is currently developed in the Roadmap deliverables (for more information refer to https://www.easa.europa.eu/domains/research-innovation/ai).
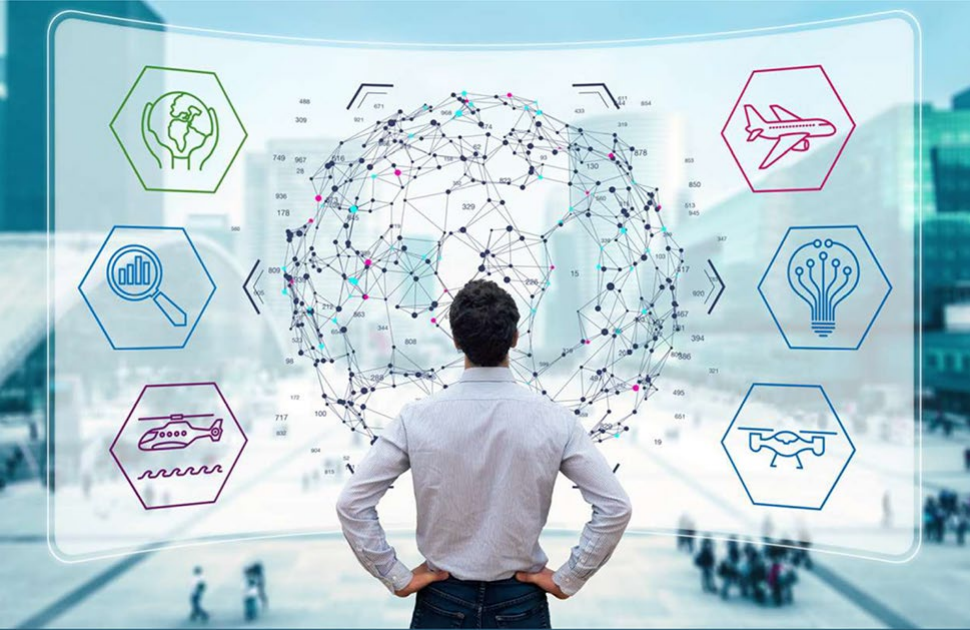


Finally, EASA is supporting the development of medium and long-term R&I agendas for aviation and air transport through its participation in the Advisory Council for Aviation Research and Innovation in Europe (ACARE), a forum which provides the framework to consolidate the varying needs and perspectives for Research and Innovation activities.

## The innovative concepts of future aircraft (H2 propulsion, electric engines, airframe design, aerodynamics, etc.) and the expected proliferation of new airborne vehicles (drones, eVTOL vehicles, etc.) imperatively raises the need for a more agile regulation framework: regulatory updates, innovations in the field of certification. How is EASA approaching this challenge?

EASA faces new societal, environmental, and technological transformations. New technologies, trends and concepts are emerging at an unprecedented pace. This requires adapting competencies, methodologies and processes to accompany those transformations. As innovations and developments are not often aligned with existing rules, rules need to evolve to enable innovation without compromising on safety. In an environment where innovation develops rapidly, we must evolve from a classical prescriptive mindset to a more performance-based approach, often based on disruptive Concepts of Operations.

Here is an example: the European and national R&I programmes, Clean Aviation and SESAR 3 are developing new aviation concepts and solutions for green technologies and sustainable fuels, which will need to be certified or approved prior to entering operation in Europe and in third countries. Furthermore, new entrants, particularly in the drones’ sector, bring new design and operational concepts to the European aeronautics arena, which necessitate new European regulatory responses. To this end the Agency also promotes innovation partnership contracts with industry. These offer a unique opportunity to learn from each other by developing common knowledge that will ease future regulatory developments while de-risking industry disruptive concepts and offering suitable certification or approval bases.

In line with its safety and environmental protection missions, EASA supports and fosters research and innovation to remain at the forefront of conceptual and technological developments and play a leading role in R&I. This includes launching and funding research projects and keeping ties with academia and institutions which can support the Agency in facing new challenges and accompanying transformations.

## How is that organised?

The EASA Research and Innovation Committee (RIC) is responsible for steering the Agency in these domains. The Committee is composed of representatives from senior management of strategic and operational Directorates. The Committee has a key role as decision-making body to achieve the following four strategic objectives:

- Build a strong partnership with key players in R&I to facilitate the introduction of new technologies and innovation;
- Provide an agile and effective regulatory system for smooth and timely integration of new technologies and innovative operations;
- Strengthen the Agency’s capacity to certify new technologies and effectively oversee innovative operation and new business models; and
- Accompany research and innovation developments through forward-looking competency management by Human Resources.

Within these parameters, the RIC engages in planning actions to reach specific objectives, oversees the Agency’s involvement in EU R&I programmes and the related partnerships with stakeholders.

Besides this, our recently established Scientific Committee supports the Agency evolution by providing technical and scientific advice on challenges faced by the aviation community with the advent of new and disruptive technologies and concepts like eVTOLs, drones, U-Space, batteries and Hydrogen electrical propulsion, and related societal transformation.

Research actions are an integral part of the European Plan for Aviation Safety (EPAS https://www.easa.europa.eu/domains/safety-management/european-plan-aviation-safety). 
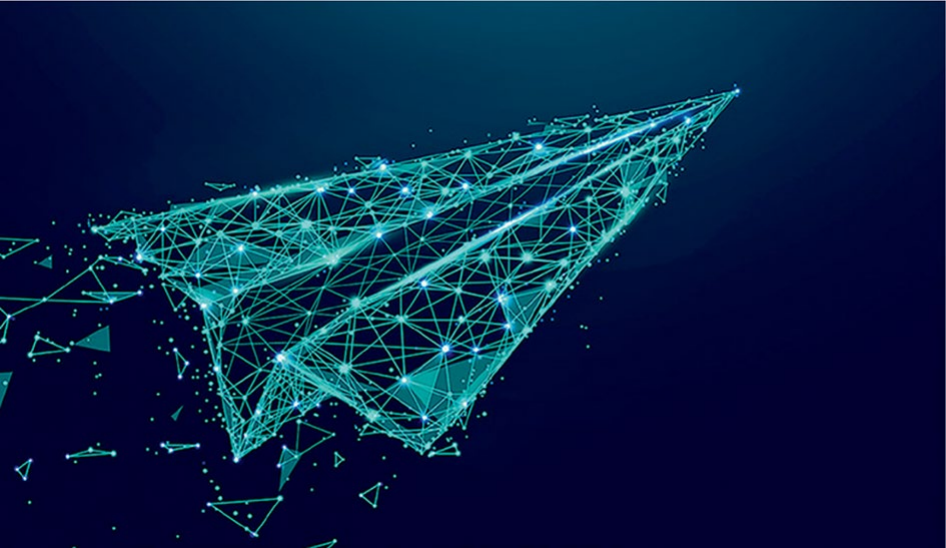


## In conclusion, what is your general feeling about the evolution of R&I and safety in aviation within the next decade?

Research and Innovation is part of aviation’s DNA. No industry has been so closely tied to the concept of innovation since its inception than aviation.

EASA manages and participates in a growing number of R&I projects to continuously improve aviation safety, but within the next decade the safety of aviation cannot be disconnected from security, health, and environmental protection. The challenge is to have an integrated approach, support technological developments and remove bottlenecks trough adapting the regulatory framework.

Our growing role in research offers huge opportunities for both the Agency and our stakeholders, including States and Industry, to be ready for the future while increasing the high level of safety reached by the aviation system. Research and Innovation is an investment in our future. EASA is fully engaged in making not only the future of aviation but also the future of our society smarter and more sustainable, to continue creating growth, wellbeing and jobs. 
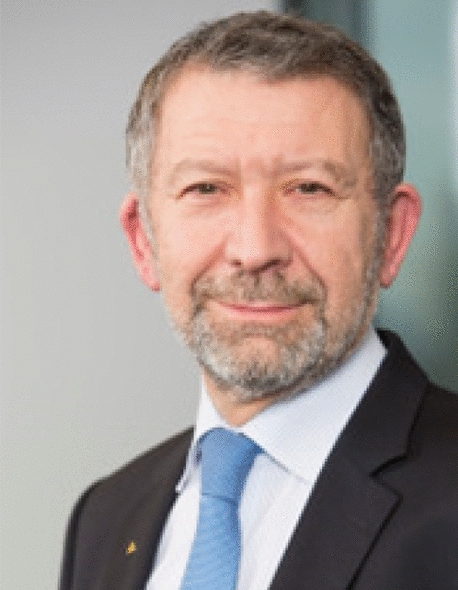


**LUC TYTGAT** **,** **EASA**

Director, Strategy and Safety Management Directorate.

Luc Tytgat has been Director of the Strategy and Safety Management Directorate of the European Aviation Safety Agency (EASA) since 1 January 2015.

He is in charge of raising safety intelligence as one of EASA’s key priorities and developing a better and more agile regulatory framework. His responsibilities also include the key strategic challenges faced by the sector such as sustainable aviation and the emerging risks: cybersecurity, conflict zones, and health and wellbeing notably COVID-19 related.

Prior to joining EASA, Luc Tytgat was Director of the Pan-European Single Sky Directorate at EUROCONTROL since 2011, after having worked for 20 years in the field of air transport and space at the European Commission and 10 years at the Belgian Air Force.

Luc Tytgat has extensive experience in dealing with European Commission matters, including air transport, Single Sky and space domains. In this context, he was responsible for the Single European Sky regulatory package, which led to the creation of the European Network Manager and an economic regulator function for ATM. He also initiated the GALILEO programme and established EU competence in the space policy sector.

